# Synergistic Tumor Cytolysis by NK Cells in Combination With a Pan-HDAC Inhibitor, Panobinostat

**DOI:** 10.3389/fimmu.2021.701671

**Published:** 2021-08-31

**Authors:** Lukman O. Afolabi, Jiacheng Bi, Xuguang Li, Adeleye O. Adeshakin, Funmilayo O. Adeshakin, Haisi Wu, Dehong Yan, Liang Chen, Xiaochun Wan

**Affiliations:** ^1^Guangdong Immune Cell Therapy Engineering and Technology Research Center, Center for Protein and Cell-Based Drugs, Institute of Biomedicine and Biotechnology, Shenzhen Institutes of Advanced Technology, Chinese Academy of Sciences, Shenzhen, China; ^2^University of Chinese Academy of Sciences, Beijing, China; ^3^CAS Key Laboratory of Quantitative Engineering Biology, Shenzhen Institute of Synthetic Biology, Shenzhen Institutes of Advanced Technology, Chinese Academy of Sciences, Shenzhen, China; ^4^Department of Stomatology, Shenzhen University General Hospital, Shenzhen University Clinical Medical Academy, Shenzhen, China

**Keywords:** HDAC, natural killer cells, cytotoxicity, chemotherapy, anti-PD-L1 therapy, immunomodulator

## Abstract

Histone deacetylases (HDAC) are frequently overexpressed in tumors, and their inhibition has shown promising anti-tumor effects. However, the synergistic effects of HDAC inhibition with immune cell therapy have not been fully explored. Natural killer (NK) cells are cytotoxic lymphocytes for anti-tumor immune surveillance, with immunotherapy potential. We showed that a pan-HDAC inhibitor, panobinostat, alone demonstrated anti-tumor and anti-proliferative activities on all tested tumors *in vitro*. Additionally, panobinostat co-treatment or pretreatment synergized with NK cells to mediate tumor cell cytolysis. Mechanistically, panobinostat treatment increased the expression of cell adhesion and tight junction-related genes, promoted conjugation formation between NK and tumor cells, and modulates NK cell-activating receptors and ligands on tumor cells, contributing to the increased tumor cytolysis. Finally, panobinostat therapy led to better tumor control and synergized with anti-PD-L1 therapy. Our data highlights the anti-tumor potential of HDAC inhibition through tumor-intrinsic toxicity and enhancement of NK –based immunotherapy.

**Graphical Abstract:**
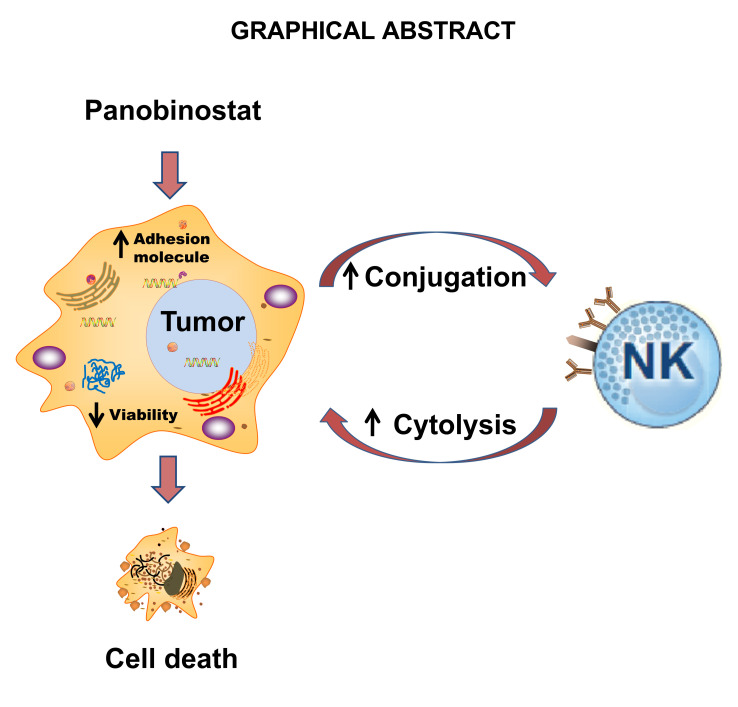


## 1 Introduction

Natural killer (NK) cells are cytotoxic components of the innate immune system and are the first line of host anti-tumor immune surveillance (1). Aside from being an early IFN-γ producer (2), the cytolytic effector activity of NK cells is an important factor that inversely correlates with tumor incidence (3). The cytolytic activity of NK cells depends on the recognition and release of perforin and granzymes into tumor cells (4–6). Recent studies on NK cell adoptive transfer therapy and NK cell checkpoint blockade have shed light on the potential of NK–based immunotherapy (7–10), whose potentials, however, have not been fully exploited. Studies have also shown that pretreatment of tumor cells with reagents could facilitate tumor cell lysis by NK cells (11–14), suggesting that tumor cells develop intrinsic mechanisms that could resist their cytolysis by NK cells.

Histone deacetylases (HDACs) belong to a family of 18 proteins (HDAC1–11 and SIRT1–7) known to deacetylate histones and non-histone proteins (15). Over-expression of HDACs is usually linked with metastatic tumors and poor prognosis (16). Histone deacetylase inhibitors (HDACi) are novel chemotherapeutic agents capable of inducing hyperacetylation of histones, leading to an altered expression (epigenetic modification) of genes controlling cell growth, cell-cycle arrest, cell differentiation, apoptosis, and inhibition of angiogenesis (17–19). Panobinostat is a novel pan-HDAC inhibitor reported to have tumor-specific cytotoxicity (20, 21). Additionally, panobinostat could facilitate antibody-dependent cell-mediated cytotoxicity effects (22), and the *in vivo* inhibition of HDAC activity does not impair NK cell functions (23); thus, these studies shed light on the potential of panobinostat in combination with immunotherapies.

Here, we assessed the cytotoxicity of panobinostat against tumor cell lines both alone and in combination with NK cells. We demonstrated the anti-tumor activities of panobinostat on human skin carcinoma (A375), cervical carcinoma (HeLa), hepatocellular carcinoma (HepG2), and hepatocarcinoma (Huh7) cell-lines with its ability to synergize with and further enhance NK cell cytolysis. Therefore, our results support the immunomodulatory efficacy of panobinostat in the treatment of tumors.

## 2 Materials and Methods

### 2.1 Cell Lines and Culture

NK (YTS) (clonal human NK cell line) and RMA-S (MHC class I-deficient variant of RBL-5) cell lines were preserved in-house. A375, HeLa, HepG2, Huh7, B16F10, and CT26 cell lines were all obtained from ATCC; HPDE, LO-2, HK-2, and MCF10A cell lines were used as control normal cells. The YTS and RMA-S cell lines were grown in RPMI-1640 (Corning) medium, HPDE was grown in DMEM/F12 + endothelial growth factor (EGF) while A375, HeLa, HepG2, Huh7, LO-2, HK-2, MCF10A, and CT26 cell lines were cultured in DMEM medium. All media were supplemented with 10% heat-inactivated fetal bovine serum (FBS), 100 U/ml of penicillin, and 100 µg/ml of streptomycin (Gibco-BRL, Gaithersburg, MD), 2mM L-glutamine as described (5, 24) and were incubated at 37°C with 5% CO2.

To obtain mouse primary NK cells, mice splenocytes were sorted. For the primary human NK cells: fresh blood was obtained from healthy donors with informed consent in compliance with the approval from the ethics committee and Institutional Review Board of Shenzhen Institutes of Advanced Technology. Peripheral blood mononuclear cells (PBMC) were isolated using Ficoll-Hypaque density gradient centrifugation. For NK cell activation and expansion, the PBMC was cultured for 21 days using the TBD™NK2013-S (Hao Yang Biological Manufacture CO., LTD, China) Cell Activation and Expansion Kit according to the manufacturer instructions.

#### 2.1.1 Drugs, Reagents, and Antibodies

Panobinostat (Cat. No.: HY-10224) and EGTA (Cat. No: HY-D0861) were obtained from MedChemExpress (Shanghai, China), DMSO (Sigma-Aldrich, D2650), InVivoMAb anti-mouse NK1.1 (clone PK136. BioXCell BE0036-5), InVivoMAb Mouse IgG2a, κ (clone C1.18.4, BioXCell BE0085). InVivoMab anti-mouse PD-L1 (clone 10F.9G2, BioXCell BP0101).

**Flow cytometry antibodies**: all antibodies used were obtained from BioLegend unless otherwise indicated. Anti-human antibodies used were: PE Fas-L (306406), PE-NKG2D (320806), PC5.5-CD11C (301624), APC-CD28 (983406), APC-CD56 (362504), PE-CD69 (310906), APC-CD80 (305219), APC-CD86 (374207), PE-CD107A (328607), PE-CD112 (337410), APC-CD226 (338311), FITC-CD3 (317305), PC5.5-CD19 (302229), PE-CD16 (302007), PE-NKp46 (331907), PE-NKp44 (325107), PE-NKp30 (325207), PE-NKG2A (375103).

Anti-mouse antibodies: FITC-CD3ϵ (145-2C11), BV-510 -CD45 (103138), PE-CD45 (103106), APC-NK-1.1 (Clone PK136) (108710), BV-510 -NK-1.1 (Clone PK136) (108737), PE-CD69 (104507), PE-CD107A (121612), PE-NKG2D (115606), APC-CD226 (128809), PE- Ly-49C/F/I/H (108207), APC-NKG2A (142807), PE- PD-1H (159604). Isotype control antibodies: APC-Rat IgG1 (401904), PE-Rat IgG1 (401906), BV-Rat IgG1 (401911).

### 2.2 Apoptosis Detection

Cell apoptosis was evaluated using Pacific Blue™ Annexin V Apoptosis - 7-aminoactinomycin D (7-AAD) Detection Kit (640926, BioLegend, San Diego, CA). The assay was performed as recommended by the manufacturer. Cells were analyzed by flow cytometry, and the gating was done to show viable cells, apoptotic cells and excluded cellular debris.

### 2.3 Real-Time *In Vitro* Proliferation Assay

The xCELLigence RTCA SP System (ACEA Biosciences Inc.) was used to assess the real-time analysis of the cellular response of A375, HeLa, HepG2, and Huh7 cell lines (25–27) to panobinostat treatment. 50ul of complete media was used to blank the 96X E-Plates, and thereafter, 10^4^ cell/50ul (per well) were seeded into the 96X E-Plates and monitored with impedance-based xCELLigence System until a log growth forming a monolayer is obtained (approximately 18-24 hours). Increasing concentration of panobinostat (25, 50, and 100nM) or DMSO control was then added into each well to make 200ul complete media. The cell index (CI) was measured continuously following ≥70 hours of incubation. CI was normalized at the end of the experiment, and it represents several cellular indicators such as cell-cell attachment, cell number, or cell size, and increased CI indicates cell growth.

### 2.4 *In Vitro* NK Cell Cytolysis

NK cell cytotoxicity against the target tumor cells was performed using the xCELLigence real-time cell analyzer (RTCA) as described previously (28). To evaluate the synergistic *in vitro* cytolytic effect of panobinostat and NK cell on the tumor cells, target cells (A375, HeLa, HepG2, and Huh7) were assessed under different conditions: (i) without panobinostat and NK cells, (ii) panobinostat only, (iii) NK cell only (iv) panobinostat and NK cells (co-treatment). For this assay, NK cell was directly added into the panobinostat treated tumor cells.

To show NK cytolysis of panobinostat and vehicle-only treated tumor cells, the target cells were pretreated with 50nM of panobinostat or vehicle for 12-18 hours; the treatment media was gently removed and replaced with complete media. Cultured human primary NK cells, NK (YTS) or primary mouse NK cells were directly added to the pretreated target cells at different effector: target ratios (E: T). To rule out the possibility of lymphocytes contributing to impedance in the cytolytic assay, sorted B cells were added to the target cells; NK cells only were also seeded to wells without target cells. Cell index for individual wells was continuously measured every 15 minutes for the specified number of hours after effector cell addition. The Cell Index (CI) was normalized using the RTCA software Pro (version 2.3.0) to remove any well-well variations, and percentage cytolysis was calculated at the end of the experiment as:

$$\%\text{ Cytolysis}=((\text{C}{\text{I}}_{\text{no effector}}-\text{C}{\text{I}}_{\text{effector}})/(\text{C}{\text{I}}_{\text{no effector}}))\times 100$$

Each cytolysis was performed in three independent experiments at four or more replicates at various effector-to-target (E: T) ratios.

To investigate the role of panobinostat-induced cell adhesion and tight junction-related gene expression in tumor cells relative to their enhanced NK cytolysis, 0.5mM EGTA (ethylene glycol tetraacetic acid) was added to block calcium-dependent adherence mediated cytolysis.

EGTA was not toxic to both target and effector cells and was used in NK cytotoxicity experiments to determine the function of adherence-conjugate mediated increased tumor cell death.

### 2.5 RNA Sequencing and Gene Set Enrichment Analysis (GSEA)

A375 cells were treated with 50nM of panobinostat or DMSO for 24 hours, the cells were wash and suspended in Trizol. Cells were then sent to GENEWIZ, Inc. (Suzhou, China) for RNA isolation, library construction, and RNA sequencing. Gene set enrichment analysis based on KEGG pathways was based on significantly differentially expressed genes.

#### 2.5.1 Reverse Transcription and Quantitative Real-Time PCR

Total RNA was extracted with TRIzol (Invitrogen), and 1μg RNA was reverse-transcribed using PrimeScript™ RT Master Mix (Cat. # RR036A, Clontech Takara Bio). Quantitative Real-Time PCR (qPCR) was performed using SYBR Green qPCR Premix Ex Taq II (Tli RNaseH Plus) (Cat. # RR820A, Clontech Takara Bio) in CFX96 Touch™ Real-Time PCR Detection System (Bio-Rad) using gene-specific primers related to the top differentially expressed Adhesion molecules and Tight junction related gene sets from the GSEA (**Supplementary Tables 1A, B**).

### 2.6 NK Cell-Target Cell Conjugation Assay

Conjugation formation between NK (YTS) cell, A375, and Hela cells was performed as described (29). Target cells were exposed to 50nM of panobinostat for 24 hours. The cells were harvested and washed with PBS to remove the drug. The treated target cells and NK (YTS) cells were then stained with CFSE and anti-human CD56-APC antibody (362504, Biolegend), respectively. The stained cells were co-incubated at 37^0^ C for the indicated time at a 1:2 effector-target ratio. This was immediately followed by fixing the cells using True-Nuclear™ Transcription Factor Buffer (BioLegend). Samples were analyzed by CytoFLEX LX Flow Cytometer (Beckman Coulter) and conjugates formed are indicated by double-positive signals for CFSE and CD56-APC.

### 2.7 Assessment of Surface Receptors on NK and Tumor Cells

To assess the cellular effect of panobinostat on modulating the surface expression of receptors and ligands on primary human NK cells, NK(YTS) and tumor cells, this was done as earlier described (28). Briefly, NK cells and tumor cells were pretreated with panobinostat or vehicle control for 12-18 hours, washed twice with PBS, and resuspended in cell staining buffer. This was followed by cell staining with specific fluorophore-conjugated antibodies or their IgG controls for 30 minutes at 4^0^C, washed again, and then directly analyzed by FACS. To analyze splenic NK cells, splenocytes were prepared from spleens obtained from panobinostat treated and control tumor-bearing mice. Splenocytes were washed and resuspended in cell staining buffer. The cells were labeled with the specific fluorophore-conjugated anti-mouse antibodies. Data was acquired using CytoFLEX LX Flow Cytometer (Beckman Coulter) and analyzed using FlowJo software (version 10).

### 2.8 Mice

Six to eight-week-old BALB/c and C57BL/6 mice were used in this study. Animal handling and the experimental procedures followed the guidelines and approved protocols of the Shenzhen Institute of Advanced Technology (SIAT), Chinese Academy of Sciences (CAS).

#### 2.8.1 *In Vivo* Experiments

BALB/c and C57BL/6 mice were subcutaneously injected with CT26 (2 X 10^5^ cells/mice), and B16F10 (2 X 10^5^ cells/mice), RMA-S (4 X 10^5^ cells/mice) cells respectively. Therapy commenced two days post-tumor challenge, and the mice were randomly divided into two groups (6 mice per group). Mice were administered with panobinostat (10 mg/kg) three times a week or PBS (untreated group) intraperitoneally (i.p). Tumor size and body weight were recorded every two days, and all mice were sacrificed when tumor volume reached 1500 mm^3^.

For NK cell depletion, mice received 200 μg mAb to NK1.1 (PK136) or Isotype control one day before tumor challenge, followed by panobinostat therapy post-tumor challenge. For the combination therapy, mice received 200 μg/ml anti-PD-L1 (clone 10F.9G2) intraperitoneally at days 3, 6, and 9. Other groups received panobinostat only, panobinostat and PD-L1, and PBS.

### 2.9 Statistical Analysis

All experiments were performed at least three independent times. Data were analyzed with GraphPad Prism9 software.

## 3 Results

### 3.1 HDACi (Panobinostat) Induced Apoptosis in Tumor Cells

**Figure 1:**
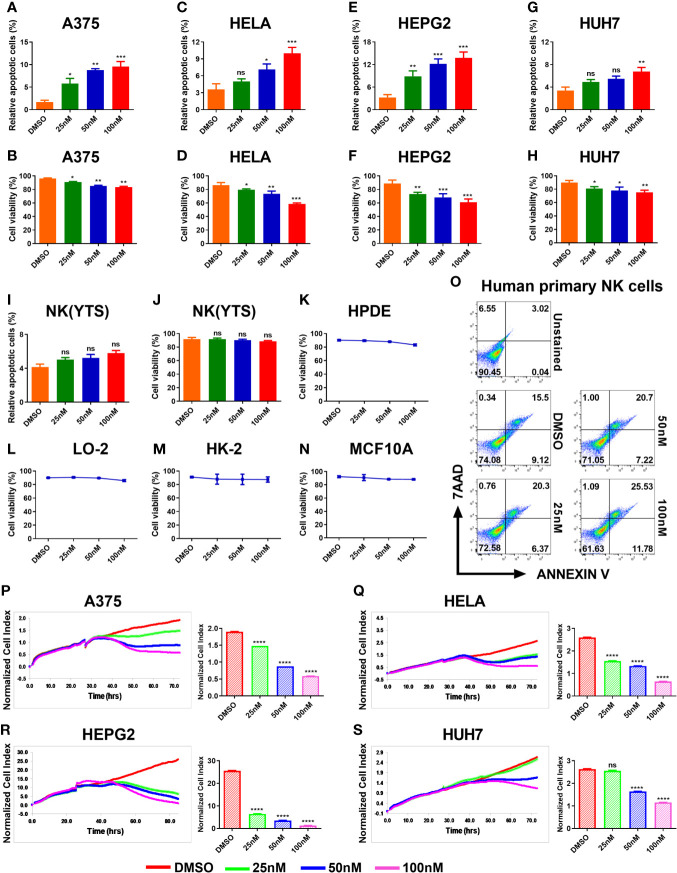
HDACi (Panobinostat) showed anti-tumor and anti-proliferative activity on tumor cells but has minimal effect on the viability of NK(YTS), normal human cell lines and primary human NK cells. Assessment of the *in vitro* panobinostat treatment on tumor cell viability. **(A, C, E, G)** apoptotic tumor cells; **(B, D, F, H)** viable tumor cells after 24 hours of increasing dose of panobinostat treatment with DMSO controls. **(I, J)** Evaluation of *in vitro* panobinostat treatment on NK (YTS), **(K–N)** normal human cell lines, and **(O)** primary human NK cells viability. Proliferation analysis of tumor cell growth treated with an increasing dose of panobinostat and DMSO control for the specified number of hours using the REAL-TIME Cell Analyzer (RTCA). **(P–S)** real-time proliferation profiles and statistical analysis of the normalized Cell Index (CI) for each proliferation profile: (CI) represents several cellular parameters, such as cell number, cell size, and cell-cell attachment; increased CI value indicates cell growth. **(A, B, P)** human skin cell carcinoma A375, **(C, D, Q)** human cervical carcinoma HeLa, **(E, F, R)** human hepatocellular carcinoma HepG2, **(G, H, S)** human hepatocarcinoma Huh7, **(I, J)** clonal human NK cell line YTS, **(K)** Human Pancreatic Duct Epithelial, **(L)** Human Hepatic Cell Line, **(M)** Human Kidney cell line, and **(N)** Human Mammary Epithelial Cells. **(A–S)** Data represent mean ± SD (n = 4 replicates) from three independent experiments. Statistical significance was assessed by t-test. (not significant ns P > 0.05, *P ≤ 0.05, **P ≤ 0.01, ***P ≤ 0.001, ****P ≤ 0.0001).

In order to investigate the tumor –suppressive effects of HDACi panobinostat (PANO) on tumor cells, we exposed the tumor cell lines to increasing concentrations of panobinostat. Nanomolecular concentration of panobinostat was sufficient to initiate apoptosis in A375 cells (**Figure 1A**), and the number of apoptotic cells increased in a dose-dependent manner compared with vehicle-treated control. In contrast, panobinostat concentrations of 50nM and 100nM induced apoptosis in HeLa cells (**Figure 1C**), while panobinostat induced a similar response in HepG2 as it did in A375 cells (**Figure 1E**). Conversely, a higher panobinostat dose of 100nM was required to induce apoptosis in Huh7 cells (**Figure 1G**). All the treated tumor cells showed an increasing percentage of apoptotic cells in a dose-dependent manner with an accompanying decrease in their cell viability (**Figures 1B, D, F, H**) compared with their respective control.

#### 3.1.1 Low Doses of HDACi (Panobinostat) Had Minimal Effect on Natural Killer (NK) and Normal Human Cell Lines Cell Viability

We further evaluated the effect of panobinostat treatment on NK(YTS) cells, four normal human cell lines, and human primary NK cells viability. NK(YTS), HPDE, LO-2, HK-2, MCF10a, and human primary NK cells were exposed to increasing doses of panobinostat for 24 hours, and their cell viability was assessed. As shown in **Figures 1I, J**, panobinostat doses (25, 50, and 100nM) tested–the doses sufficient to induce apoptosis in all the other tumor cells–failed to affect NK(YTS) cell viability significantly. Similarly, the viability of the four normal cell lines and human primary NK cells tested showed minimal effect on their viability upon panobinostat treatment (**Figures 1K–O**).

#### 3.1.2 Growth Inhibitory Effect of HDACi (Panobinostat) on Tumor Cells

The data from the x-CELLigence system showed that panobinostat significantly inhibited proliferation in all the treated tumor cells in a dose-dependent manner in comparison with the control (**Figures 1P–S**). Real-time proliferation profiles show that panobinostat had a significant inhibitory effect on A375, HeLa, and HepG2 cells compared to vehicle-only treated cells (**Figures 1P–R** respectively). However, only the 50-100nM of panobinostat significantly affected Huh7 cell proliferation (**Figure 1S**).

### 3.2 Pretreatment of Tumors Cells With HDACi (Panobinostat) Enhances Their NK Cell Cytotoxicity

**Figure 2:**
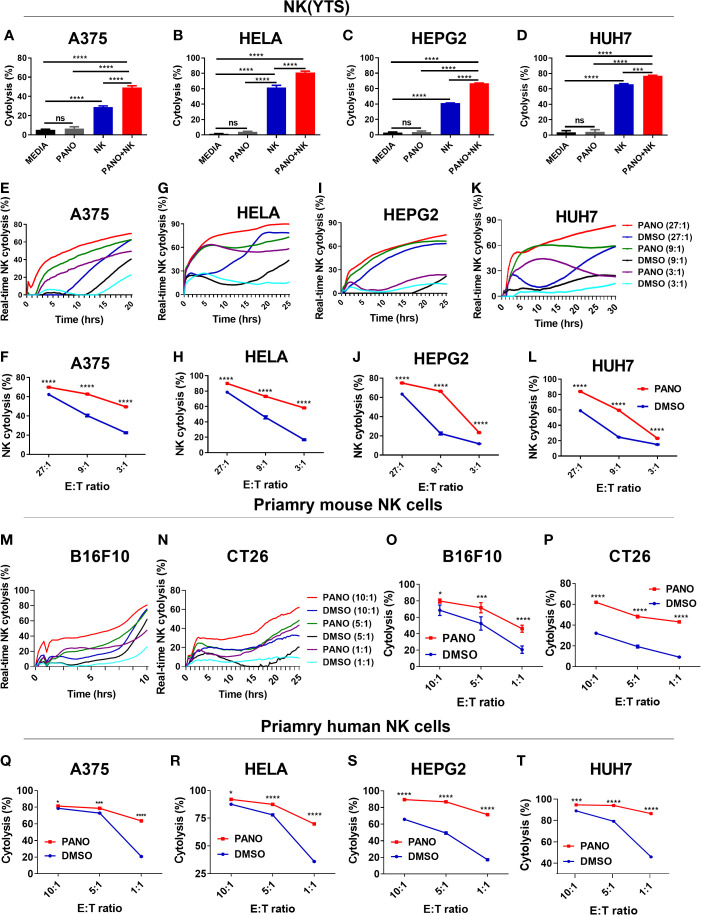
Pretreatment of tumor cells with HDACi (Panobinostat) increase their susceptibility to NK cell cytolysis. Assessment of the synergistic *in vitro* cytolytic effect of co-treatment of panobinostat and NK cell on tumor cells **(A–D)**. Evaluation of the *in vitro* NK cell cytolysis of panobinostat (50nM) or vehicle pretreatment of tumor cells for 12-18 hours prior to NK cell cytolysis; **(E, G, I, K)** real-time NK cell cytolysis using the REAL-TIME Cell Analyzer (RTCA); **(F, H, J, L)** NK cell cytolysis of tumor cells at different E: T ratio. **(E, F)** human skin cell carcinoma A375, **(G, H)** human cervical carcinoma HeLa, **(I, J)** human hepatocellular carcinoma HepG2, **(K, L)** human hepatocarcinoma Huh7. Primary mouse NK cell cytolysis of mouse tumor cells **(M–P)**; **(M, N)** real-time NK cell cytolysis. **(Q–T)** Primary human NK cell cytolysis of tumor cells at different E: T ratio. **(A–D)** Percentage cytolysis is expressed as the mean value ± SD (n = 4 replicates); **(F, H, J, L, O–T)** Percentage NK cell cytolysis is expressed as the mean value ± SD (n = 4 replicates) for each E: T ratio from three independent experiments. Statistical significance was assessed by t-test **(A–D)** and two-way ANOVA **(F, H, J, L, O–T)** (not significant ns, P > 0.05, *P ≤ 0.05, ***P ≤ 0.001, ****P ≤ 0.0001).

A critical part of any successful chemotherapeutic regime is its ability to stimulate an endogenous anti-tumor response. Certain chemotherapeutic drugs have been reported to sensitize tumor cells to immune effector cell cytolysis (30). Low doses of chemotherapeutic drugs have also been reported to have immunomodulatory effects (31). To establish the synergistic *in vitro* cytolytic effect of panobinostat and NK cell on tumor cells, we first assessed the target cells under the different condition: (i) without panobinostat and NK cells, (ii) panobinostat only, (iii) NK cell only, and (iv) panobinostat and NK cells. We observed that tumor cells co-treated with panobinostat and NK cell showed a synergistic increased cytolytic effect compared to panobinostat or NK cell treatment only (**Figures 2A–D**).

We also evaluated the effect of panobinostat (50nM) or vehicle pretreatment of tumors prior to NK(YTS) cytolysis. As shown in **Figures 2E, F**, panobinostat pretreated A375 tumor cells showed increased NK cytolysis compared to vehicle control for the different E: T ratios. Similarly, panobinostat pretreated HeLa cells (**Figures 2G, H**), HepG2 cells (**Figures 2I, J**), and Huh7 cells (**Figures 2K, L**) all showed a significant increase in NK cell-mediated cytolysis.

We further evaluated the cytolysis of panobinostat treated tumor cells against primary mouse NK cells (M-P) and human NK cells (Q-T). The data showed that panobinostat pretreated B16F10 and CT26 mouse tumor cells (**Figures 2M–P**) had increased NK cytolysis compared to the controls. Similarly, panobinostat pretreated human tumor cell lines (**Figures 2Q–T** and **Supplementary Figures 1A–D**) had increased NK cytolysis compared to their respective controls. These data suggest that panobinostat pretreated tumor cells are highly susceptible to NK cell cytolysis.

### 3.3 HDACi (Panobinostat) Suppresses Tumorigenesis Gene Sets and Promotes Cell Adhesion and Tight Junction Genes

**Figure 3:**
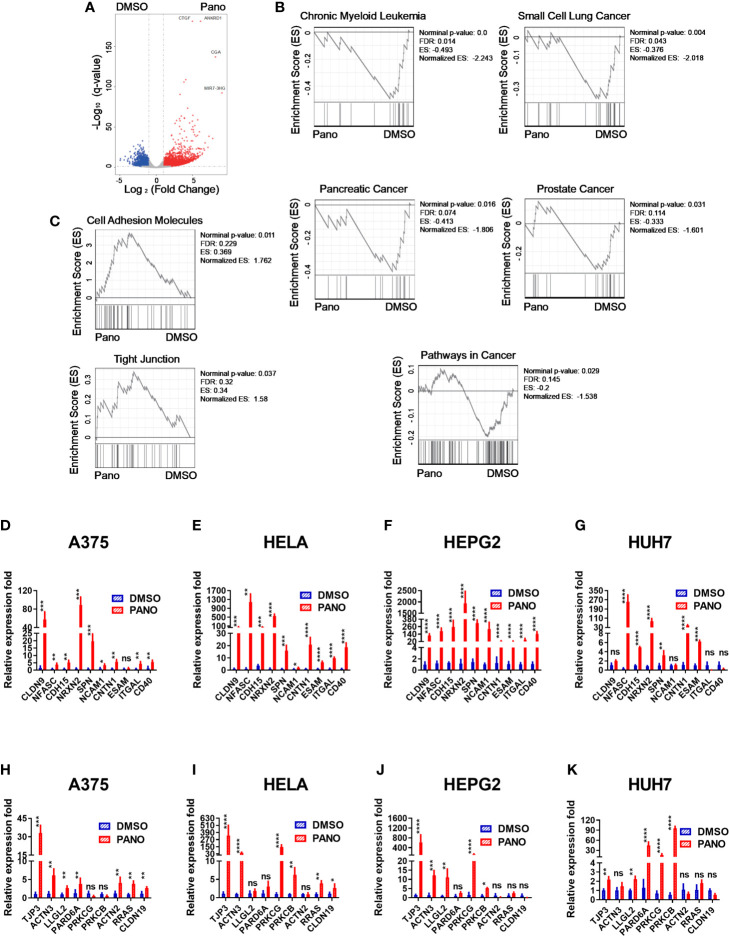
Transcriptome analysis of A375 cells treated with HDACi (Panobinostat) and RT-qPCR validation of cell-adhesion and tight junction gene expression from RNA-seq. A375 cells were treated with 50nM Panobinostat and DMSO control for 24 hours prior to RNA isolation and transcriptome analysis (n = 3). **(A)** Differentially expressed genes were shown. **(B, C)** Gene sets enriched in either the DMSO or Panobinostat groups. **(D–K)** The expression levels for the top ten differentially expressed genes from the cell-adhesion and tight junction related genes were assessed using the RT-qPCR relative to the expression of Beta Actin. **(D–G)** cell-adhesion gene sets, **(H–K)** tight junction gene sets. **(D, H)** human skin cell carcinoma A375, **(E, I)** human cervical carcinoma HeLa, **(F, J)** human hepatocellular carcinoma HepG2, **(G, K)** human hepatocarcinoma Huh7. Relative expression fold is expressed as mean ± SD (n = 4 replicates) from three independent experiments. Statistical significance was assessed by t-test **(D–K)** (not significant ns, P > 0.05, *P ≤ 0.05, **P ≤ 0.01, ***P ≤ 0.001, ****P ≤ 0.0001).

In order to provide mechanistic insights into the increased apoptosis of panobinostat–treated tumor cells, as well as their increased NK cell killing, we performed transcriptome analysis on panobinostat or DMSO–treated A375 cells. Panobinostat treatment resulted in a number of differentially expressed genes (DEGs) (**Figure 3A**). Gene sets enrichment analysis (GSEA) showed that some gene sets in cancers were significantly enriched in the DMSO control group (**Figure 3B**), indicating that panobinostat decreased gene expression involved in tumorigenesis; which might account for the decreased survival and/or increased apoptosis we observed in panobinostat–treated tumor cells.

Furthermore, we found that panobinostat treatment caused the enrichment of Cell Adhesion Molecules and Tight Junction gene sets (**Figure 3C**) in the treated group. The enriched gene sets were further confirmed by RT-qPCR on all the treated target tumor cells (**Figures 3D–K**). There was significant upregulation of Cell Adhesion Molecules genes (**Figures 3D–G**) and Tight Junction genes (**Figures 3H–K**).

Since NK cell cytolysis against tumor cells requires conjugate formation between NK cells and tumor cells, the enriched genes of cell adhesion might explain the increased cytolysis against these tumor cells by NK cells.

### 3.4 HDACi (Panobinostat) Increases Conjugate Formation Between NK Cells and Tumor Cells

**Figure 4:**
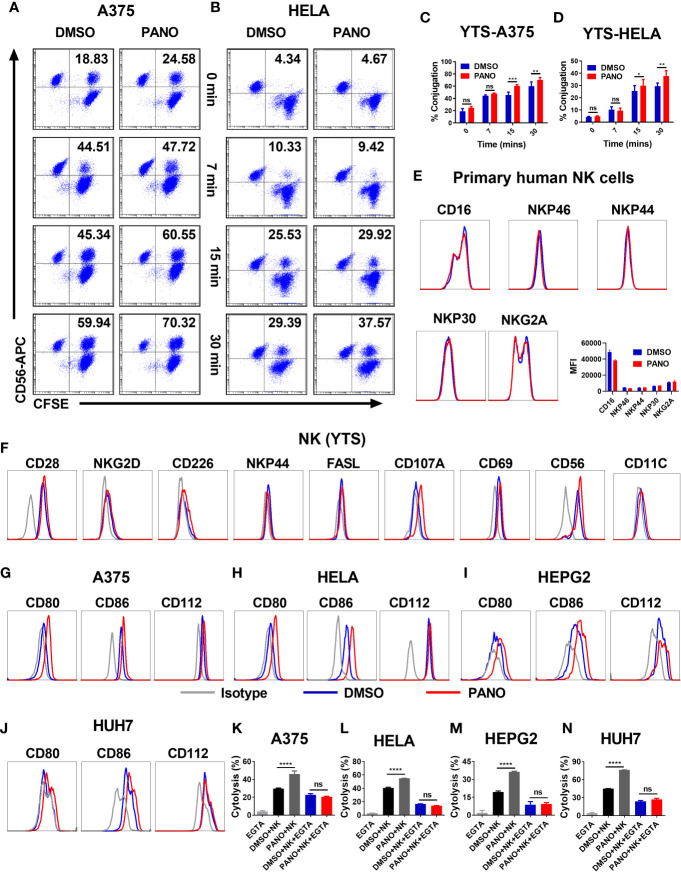
HDACi (Panobinostat) pretreatment promotes NK cell-target cell conjugation formation and modulates the expression of NK cell-activating receptors. Tumor cells were treated with 50nM Panobinostat or DMSO for 24 hours, labeled with CFSE prior to co-incubation with CD56-APC labeled NK(YTS) cells for the indicated times. Conjugates formation between NK(YTS) and target cells (**A**, **C** A375 cells) and (**B**, **D** Hela cells) are indicated as the double-positive population as assessed by flow cytometry. Assessment of primary human NK cells, NK(YTS) and Tumor cells pretreated with 50nM of Panobinostat (Red lines) or DMSO control (Blue lines) before flow cytometry assessment of the specified activating and inhibitory receptors and the corresponding IgG Isotype controls (Grey line). Assessment of surface expression pattern of selected activating and inhibitory receptors on **(E)** primary human NK cells; **(F)** NK(YTS) cells; **(G–J)** Assessment of surface expression pattern of NK cell activating ligands on tumor cells. **(K–N)** Evaluation of the *in vitro* NK cell cytolysis of Panobinostat (50nM) or vehicle pretreated tumor cells in the presence of EGTA (a divalent cation chelating agent). **(A, B)** Representative dot plots, **(C, D)** Statistical analysis of the conjugates formed at different time points. Percentage conjugation is expressed as mean ± SD (n = 3 replicates) from three independent experiments. (K–N) Percentage cytolysis is expressed as the mean value ± SD (n = 4 replicates) from three independent experiments. Statistical significance was assessed by a two-way ANOVA **(C, D)** and t-test **(K–N)**. (not significant ns, P > 0.05, *P ≤ 0.05, **P ≤ 0.01, ***P ≤ 0.001, ****P ≤ 0.0001).

NK cell cytolysis against target cells involves a multiple-step approach, including conjugation formation with its target cells *via* adhesion molecules (32). GSEA and qPCR showed the enrichment of cell adhesion molecules in panobinostat-treated tumor cells, suggesting an enhanced conjugation between NK and the target cells. Indeed, we observed significantly increased conjugation between NK and panobinostat-treated A375 tumor cells compared with the vehicle-only group after 15 minutes of co-incubation (**Figures 4A, C**). A similar trend was observed between NK and panobinostat-treated Hela cells (**Figures 4B, D**). This indicates that panobinostat promotes the conjugation formation between NK cells and tumor cells.

#### 3.4.1 HDACi (Panobinostat) Pretreatment Modulates the Expression of NK Cell Triggering Receptors on Primary Human NK Cells, NK (YTS) and Tumor Cells

Based on our observed *in vitro* findings, we sought to find more mechanistic insight into the enhancement of NK cell killing of panobinostat-treated tumor cells. Since NK cell effector functions are dictated by a balance between its activating and inhibitory receptors, the pretreatment of primary human NK cells with panobinostat or vehicle control, followed by the assessment of CD16, natural cytotoxicity receptors (NCRs)–NKp46, NKp44, NKp30 and NKG2A by FACS revealed no observable differences between the treated and control groups albeit with slight reduction in CD16 expression of panobinostat group (**Figure 4E**).

Similarly, the pretreatment of NK (YTS) cells with panobinostat or vehicle control, followed by the assessment of its surface receptors by flow cytometry, revealed no significant difference between panobinostat treatment relative to the vehicle-treated control (**Figure 4F** and **Supplementary Figure 1E**). We observed a relatively low expression of the activating surface receptors (NKG2D, CD226, NKP44, and FASL), with no significant difference between the treated and control groups. To further highlight the involvement and enhancement of effector-target conjugation in the increased tumor killing by panobinostat, we assessed the expression of CD56 and CD11C (two cell adhesion surface receptors that play an important role in cell-cell contact) on the NK (YTS) cell. Our data revealed a slight enhanced expression of CD56 surface receptors by panobinostat relative to its control (**Figure 4E**, **Supplementary Figure 1E**).

The expression pattern of CD80, CD86 (ligands for CD28), and CD112 (another activating ligand for NK cell) were assessed on all the four target cells. Our data showed a significantly higher surface expression of these ligands by panobinostat treatment relative to the control (**Figures 4G–J** and **Supplementary Figures 1F, I**). This suggests that panobinostat, in addition to increasing cell adhesion molecule expression, promotes the expression of NK cell activating ligands on tumor cells.

#### 3.4.2 Abrogation of Cell Adhesion and Tight Junction Induced NK Cell Cytolysis With EGTA (Ethylene Glycol Tetraacetic Acid)

To further strengthen the role of cell adhesion and tight junction gene expression in the enhanced NK cytolysis of the tumor cells, EGTA (chelating agent of divalent cations) was added to block the calcium-dependent adherence and conjugation formation between NK and tumor cells. As shown in **Figures 4K–N**, the observed increased NK cytotoxicity in panobinostat-treated target cells compared with the control were completely abrogated. This suggests that panobinostat promotes the conjugation formation between NK cells and tumor cells, contributing to the enhanced cytolysis.

### 3.5 HDACi (Panobinostat) Therapy Enhanced Better Control of Tumor Growth

**Figure 5:**
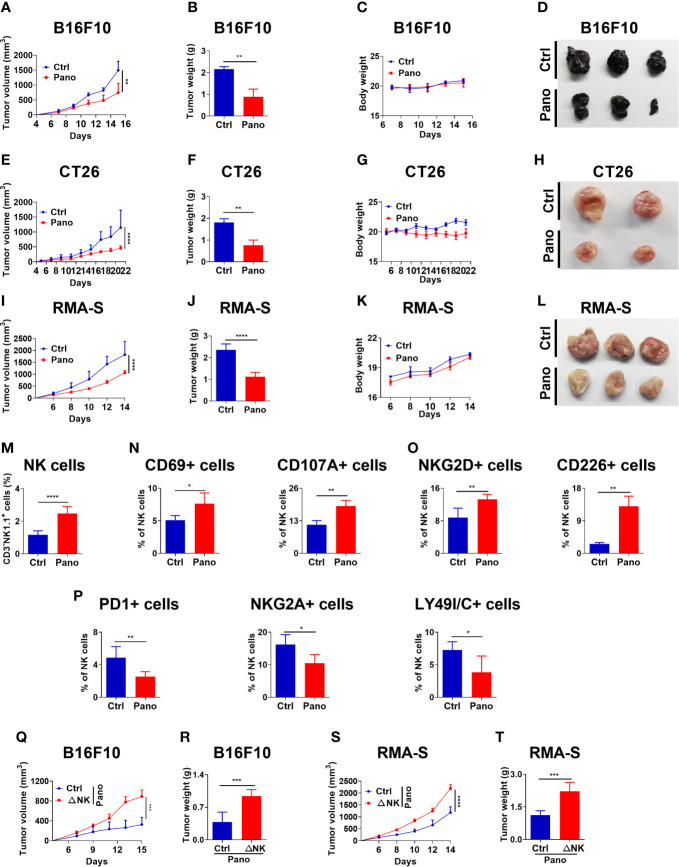
HDACi (Panobinostat) therapy enhances better tumor control *in vivo* and promotes splenic NK cell activation. Evaluation of tumor growth kinetic upon Panobinostat therapy (Tumor growth curve, Tumor weight, Bodyweight and, Tumor images at the end of the experiment). Tumor growth kinetic followed with Panobinostat treatment for **(A–D)** C57BL/6 mice inoculated with B16F10 (2 X 10^5^ cells/mice), **(E–H)** BALB/c mice inoculated with CT26 (2 X 10^5^ cells/mice), **(I–L)** C57BL/6 mice inoculated with RMA-S (4 X 10^5^ cells/mice). **(M–P)** Assessment of splenic NK cell activation, degranulation, and expression of selected surface receptors in an RMA-S tumor model. **(M)** percentage of splenic NK cells; **(N)** percentage of CD69+ and CD107A+ NK cells; **(O)** percentage of activating NK cell receptors; **(P)** percentage of inhibitory NK cell receptors. **(Q–T)** NK cell depletion (ΔNK) one day prior to tumor inoculation, followed with Panobinostat therapy. Assessment of tumor growth kinetic **(Q, R)** B16F10 and **(S, T)** RMA-S, tumor models. **(M–P)** Data are expressed as mean ± SD (n= 6 replicates) from two independent experiments. Statistical significance was assessed by an t-test. (P > 0.05, *P ≤ 0.05, **P ≤ 0.01, ***P ≤ 0.001).

We further investigated the therapeutic effect of panobinostat administration in suppressing tumor growth. First, we investigated whether panobinostat therapy could suppress the growth of MHC-I –sufficient tumors and NK –sensitive MHC-I –deficient tumor *in vivo*. Our findings showed significant tumor growth suppression in panobinostat-treated tumor-bearing mice relative to their untreated control (**Figures 5A–L**). Both the BALB/c and C57BL/6 tumor-bearing mice displayed similar tumor growth kinetics for the panobinostat treated group compared to control with little or no observable loss of body weight– indicative of the low or no systemic toxicity of panobinostat administration (**Figures 5C, G, K**). Additionally, panobinostat therapy resulted in significantly prolonged survival of the treated mice relative to the control (Supplementary **Figures 1J, K**). Our data showed that panobinostat therapy could suppress the growth of both MHC-I –sufficient and NK –sensitive MHC-I –deficient tumors *in vivo*.

#### 3.5.1 Panobinostat Therapy Promotes NK Cell Activation *In Vivo*

Sequel to our *in vivo* anti-tumor data and the *in vitro* involvement of NK cells in Panobinostat’s anti-tumor activity, we further investigated the ability of panobinostat to arouse endogenous immune cells and engage the anti-tumor activity of NK cells, we assessed splenic NK cells population from panobinostat treated RMA-S tumor-bearing C57BL/6 mice and the control. We observed a significant percentage increase of NK cells in the panobinostat group relative to the control (**Figure 5M**). The assessment of CD69 and CD107A (two surface markers that show NK cell activation and degranulation) revealed a significantly higher proportion of these activated NK cell populations in the treated group relative to the control (**Figure 5N**).

Similarly, assessment of some activating (NKG2D and CD226) and inhibitory (PD1, NKG2A, LY49I/C) surface receptors on the splenic NK cells showed that panobinostat promoted the expression of activating receptors with significantly reduced expression of the inhibitory receptors (**Figures 5O, P**). Hence, these data suggest that panobinostat therapy promotes NK cell activation to suppress tumor growth *in vivo*.

#### 3.5.2 NK Cells Are Required to Support the Anticancer Activity of Panobinostat

Since our *in vivo* data reveal that panobinostat therapy could facilitate anti-tumor immunity against different tumor models and enhance tumor cell killing *in vitro*, we hypothesized that NK cells are required for panobinostat anti-tumor activity. We investigated the contribution of NK cell to the therapeutic effects of panobinostat in C57BL/6 mice bearing B16F10 and RMA-S tumors. As expected, the depletion of NK cells by mAb one day before s.c inoculation of tumor cells in the mice followed with panobinostat treatment revealed a significant loss of the therapeutic benefits of panobinostat therapy in NK cell depleted group for both B16F10 and RMA-S in comparison to their control (**Figures 5Q–T** and **Supplementary Figures 1L-O**). Since the curative activity of panobinostat is almost completely lost upon NK cell depletion, this suggests that NK cells contributed to the anti-tumor effects of panobinostat.

### 3.6 Panobinostat Synergizes With Anti-PD-L1 Therapy

**Figure 6:**
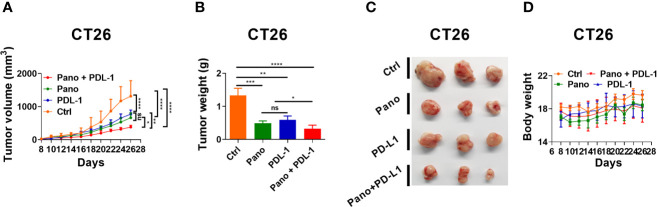
HDACi (Panobinostat) synergizes with anti-PDL-1 therapy. Evaluation of tumor growth kinetic for anti-PDL-1 and Panobinostat therapy (Tumor growth curve, Tumor weight, Tumor images, and Bodyweight at the end of the experiment). **(A–D)** Tumor growth kinetic for BALB/c mice inoculated with CT26 (2 X 10^5^ cells/mice). Statistical significance was assessed by t-test. (not significant ns, P > 0.05, *P ≤ 0.05, **P ≤ 0.01, ***P ≤ 0.001, ****P ≤ 0.0001).

The activity of NK cells is required for anti-PD-L1 immunotherapy (8). Since panobinostat could simultaneously suppress tumor growth and stimulate NK cell activity *in vivo*, we reasoned whether panobinostat could enhance anti-PD-L1 therapy. To investigate this, we administered anti-PD-L1(mAb) or a combination of anti-PD-L1 and panobinostat to CT26 tumor-bearing BALB/c mice. We observe a significant tumor growth suppression in the combination therapy relative to the control and the monotherapies of panobinostat and anti-PD-L1 (**Figures 6A–D**). These data revealed that the HDAC inhibition and immune checkpoint blockade presents a beneficial anti-tumor therapy compared to the monotherapies. Also, there were little or no changes in the mice’s body weights (**Figure 6D**), indicating the tolerability with no systemic toxicity in this combination therapy.

## 4 Discussion

Many tumor cells often develop resistance to standard chemotherapeutic drugs when conventional treatment strategies are employed, resulting in failed clinical outcomes. To address these issues, alternative approaches such as combination therapy, immunotherapy, and stimulation of endogenous anti-tumor immunity are currently being used (33). In the current study, we focused on examining the cytotoxicity of pan-HDAC inhibitor panobinostat against tumor cell lines both alone and in combination with NK cells, particularly to show its immunomodulatory potential on these tumor cell lines.

We found that panobinostat induced apoptosis in all tumor cells, with an associated decrease in their cell viability upon 24hours of treatment in a dose-dependent manner (**Figures 1A–H**). This finding is consistent with previous reports on Panobinostat’s antitumorigenic properties (21, 34–36). However, low doses (25-100nM) of panobinostat treatment of NK(YTS) cells failed to significantly affect its cell viability compared with the vehicle-treated control (**Figures 1I, J**). Similarly, panobinostat treatment of four normal human cell lines and primary human NK had a negligible effect on their viability (**Figures 1K–O**). These findings are consistent with previous studies that found that low doses of panobinostat failed to significantly affect NK cells viability (22), with selective induction of apoptosis in tumor cells but no effect on normal human cell lines (37).

Proliferative analysis of panobinostat treatment on the tumor cells showed significant inhibition of cell proliferation (**Figures 1P–S**). As little as 50nM was sufficient to induce significant inhibition of cell proliferation in all the tested tumor cells, our observation is in line with earlier reports (21, 34, 36, 38).

The use of NK cells for cancer immunotherapy has been well discussed (33, 39); NK cells are particularly effective in eliminating tumor cells *via* many mechanisms. Our findings on the synergistic cytolytic effect of panobinostat and NK cells on tumor cells compared to panobinostat or NK cell treatment alone (**Figures 2A–D**) may be due to the fact that chemotherapeutic agent can invoke different cellular responses in tumor cells, such as the induction of the stress pathways (30, 40, 41) which can serve as triggers for NK cell activation/cytotoxicity (30, 40–42). Since immunomodulators have the potential to modulate the surface expression pattern of surface activating and inhibitory ligands on tumor cells– rendering them immunogenic to NK cell cytolysis (28, 30), our data on the immunomodulatory role of panobinostat revealed that pretreatment of tumors prior to NK cell cytolysis increases their sensitization to NK(YTS) cytolysis (**Figures 2E–L**). Low doses of panobinostat treatment of the tumor cells synergize with NK cell cytotoxicity at the tested E: T ratios. Similarly, when we used primary NK cells from mice (**Figures 2M–P**) and humans (**Figures 2Q–T**), we saw increased NK cytolysis of panobinostat-treated tumor cells compared to controls. A previous study found that panobinostat treatment increased the expression of NK cell-activating ligands, which can activate NK cells and account for the increased NK cell-mediated cytolysis (43).

Our GSEA data revealed a significant enrichment of certain gene sets in cancers for the DMSO control group, which provided some mechanistic insight into Panobinostat’s anti-tumor activity (**Figure 3B**), suggesting that panobinostat repressed the expression of genes involved in tumorigenesis. This observation could account for, in part, the decreased survival and/or increased apoptosis associated with panobinostat-treated tumor cells. The identification of these cancer-related DEGs (such as those related to chronic myeloid leukemia, small cell lung cancer, pancreatic cancer, and prostate cancer) have previously been reported to regulate certain physiological processes involved in cell cycle, cell proliferation, extracellular matrix (ECM)-receptor interaction, cell adhesion, and many signaling pathways (44–51). Alterations in these DEGs have been established to affect the survival and induction of apoptotic phenotypes of tumor cells (52–54).

The transcriptome, qPCR, and conjugation assay data provide some mechanistic insight into panobinostat-treated tumor cells’ increased NK cell cytolysis. These findings revealed that genes involved in tumorigenesis were significantly reduced, whereas cell adhesion molecules and tight junction gene sets were enriched for panobinostat-treated tumor cells, as confirmed by RT-qPCR for all target tumor cells (**Figures 3D–K**). This implies that panobinostat may reduce tumor cell survival while increasing their conjugation to NK cells, thereby increasing NK cell cytolysis.

Adhesion molecules have been shown to increase tumor cell binding avidity to effector cells and are critical mechanisms for effector-tumor cell cytolysis (55). Our conjugation assay data support Jackson et al, findings that conjugate formation between lymphokine-activated killer cells and bladder cancer cell lines is a prerequisite for tumor cell cytotoxicity (56). Besides, activated NK cells have been shown to form tight conjugation with adhesion molecules on target tumor cells, resulting in increased engagement of NK cell activating receptors, which facilitate its activation and tumor cell cytotoxicity (57). Several other reports have shown the importance of tumor cell adhesion molecules in conjugation formation between effector cells and tumor cytotoxicity (55–59).

Based on the premise that NK cell activation is dependent on the balance of activating and inhibitory signals received from its surface receptors, FACS analysis of the natural cytotoxicity receptors (NCRs) on the surface of panobinostat-treated primary human NK cells showed no significant observable difference in comparison to the control. In the same vein, FACS analysis of the activating surface receptors on panobinostat-treated NK(YTS) cells revealed no significant difference in expression compared to the vehicle-treated control. Although there appears to be relatively low surface expression of other activating receptors, we found an enhanced expression of CD56 (cell adhesion surface receptors) that play an important role in cell-cell contact for panobinostat-treated NK(YTS) cells relative to control. Our data is consistent with earlier reports showing the importance of CD56 –neural cell adhesion molecule (NCAM) (60). CD56 (NCAM) expression on NK cells has been shown to induce activation signals *via* adhesion molecules, as well as an increase in cytotoxicity and phosphorylation of the human NK cell signaling cascade (60). Also, CD56 expression –similar to CD69 and HLA-DR expression –has been shown as an activation marker of NK cells with enhanced cytotoxicity (61).

Sequel to the above observation, assessment of the expression of NK cell activating ligands (CD80 and CD86, and CD112) on panobinostat-treated target tumor cells revealed an enhanced expression of these ligands relative to their controls. These findings provide a further mechanistic explanation for the increased NK cell cytolysis of panobinostat-treated tumor cells, as well as support for Panobinostat’s immunomodulatory roles. Collectively, our findings revealed that panobinostat could modulate the expression pattern of surface activating receptors on NK cells and their ligands on tumor cells, leading to enhanced tumor cell immunogenicity to NK cell cytotoxicity. These findings are consistent with our previous research on a small molecule’s ability to modulate the surface expression of activating receptors on NK cells and its ligands on tumor cells (28).

Cell adhesion molecule expression, which is responsible for cell-cell adhesion, has been shown to be calcium-dependent (62, 63). To further confirm the role of cell adhesion and tight junction gene expression in the enhanced NK cytolysis of the tumor cells, we assessed NK cytolysis of panobinostat treated tumor cells with or without EGTA. Similarly, NK-target cell conjugation requires calcium flux (64, 65); thus, the presence of EGTA (a potent chelating agent of divalent cations including Ca^2+^) abrogated the enhanced cytolysis observed for panobinostat pretreated tumor cells (**Figures 4K–N**) possibly by blocking the tight conjugation formation between NK and tumor cells as well as abolishing the granzymes/perforin exocytosis. Therefore, the elimination of enhanced NK cell cytolysis mediated by panobinostat treatment in the presence of EGTA confirms the involvement of adhesion molecules and conjugation formation between NK-target cells, lending support to Panobinostat’s potential immunomodulatory mechanism. Although panobinostat was not developed as immunostimulatory drugs. It is therefore understandable that its immunomodulatory activity depends on multiple signaling pathways that are activated on induction of sublethal damage, epigenetic modification and induction of cellular stress.

Panobinostat has largely been studied *in vitro* for its therapeutic benefit on many cancer types. Our *in vivo* data affirms the anti-tumor potential of panobinostat. The therapeutic ability of panobinostat to engage host anti-tumor immunity was demonstrated in both BALB/c and C57BL/6 tumor bearing mice (**Figures 5A–L**). Also, panobinostat treatment delayed tumor growth and prolonged overall mouse survival (**Supplementary Figures 1J, K**).

We further substantiated Panobinostat’s anti-tumor properties and its engagement of host antitumor immunity by assessing the activation status of splenic NK cells (**Figures 5M–P**), thereby strengthening its ability to augment NK cell anti-tumor functions, possibly through the recruitment of tumor-associated NK cells. The role of NK cell activating receptors in tumor and infection control is well established (8, 66, 67).

Additionally, our data showed the involvement of NK cells in the anti-tumor activity of panobinostat following the loss of the therapeutic advantage of panobinostat when NK cells are depleted (**Figures 5Q–T**, **Supplementary Figures 1A–D**)– suggesting that the *in vivo* anti-tumor therapeutic activity of panobinostat is NK cell-dependent. The administration of panobinostat is also well-tolerated since little or no loss in body weights in the mice suggests little or no systemic toxicity. Our findings corroborate previous research on the therapeutic benefit of panobinostat administration in tumor-bearing mice (22, 68, 69).

Immune checkpoint blockade is a promising therapeutic approach for various types of cancer. Despite the documented success of approved ICB therapies, such as anti-PD-L1, a subset of patients experiences failed ICB efficacy in clinics (33, 70); prompting the development of novel combinatory regimens to overcome failed clinical ICB therapies. The combination of HDAC inhibitors with ICB has been proposed as a viable treatment option (71). As demonstrated by our findings, combining panobinostat with anti-PDL-1 resulted in increased anti-tumor efficacy (**Figures 6A–D**). The improved anti-tumor efficacy demonstrated in the current study could be attributed to Panobinostat’s ability to augment MHC and antigen differentiation in tumor cells, resulting in a superior activation of an endogenous anti-tumor immune response (68). Our findings are consistent with previous studies that show the combined efficacy of HDAC inhibitors and ICB mAb (71–73). The current study highlights the importance of inhibiting HDACs in conjunction with ICB therapies for total tumor regression.

Although our current study only partly explored the underlying mechanisms of panobinostat and attributed to the upregulation of adhesion molecule and tight junction gene sets, with the upregulation of tumor specific antigens targeted by NK cells, this study is not exhaustive, it does open the door for further research into other underlying mechanisms associated with panobinostat sensitization of tumor cells to NK cell killing. To address the current issue of failed clinical outcomes from conventional chemotherapy regimens, the use of chemo-immunotherapy approaches focused on the identification of immunomodulatory agents capable of regulating tumor cell expression of immunogens while simultaneously improving the activities of cytotoxic lymphocytes (CTLs) such as NK cells for total tumor remission are required.

## Data Availability Statement

The datasets presented in this study can be found in online repositories. The names of the repository/repositories and accession number(s) can be found below: NCBI Sequence Read Archive (SRA) database (https://www.ncbi.nlm.nih.gov/sra/), accession number(s) SRX11149113 - SRX11149117 and NCBI BioProject, accession number PRJNA737652.

## Ethics Statement

The studies involving human participants were reviewed and approved by the Ethics committee and Institutional Review Board of Shenzhen Institutes of Advanced Technology, Chinese Academy of Science (CAS). The patients/participants provided their written informed consent to participate in this study. The animal study was reviewed and approved by Shenzhen Institute of Advanced Technology (SIAT), Chinese Academy of Sciences (CAS).

## Author Contributions

LA and JB designed the experiments, analyzed the data, and wrote the manuscript. LA performed the experiments. JB performed transcriptome analysis. XL contributed new reagents/analytic tools. XL, AA, FA, HW, DY, and LC validated the investigation and reviewed the final draft. XW supervised the project and oversaw the writing process. All authors contributed to the article and approved the submitted version.

## Funding

This work was funded by the National Key R&D Program of China (2019YFA0906100 and 2020YFA0710802), the Natural Science Foundation of China (82071768), Key-Area Research and Development Program of Guangdong Province (2019B020201014), the Natural Science Foundation of Guangdong Province (2019A1515011412), Shenzhen Basic Science Research Project (JCYJ20170818164619194), Shenzhen Basic Science Research Project (JCYJ20170413153158716), Special funds for major science and technology of Guangdong province (2019B020201014), and Nanshan pilot team project (LHTD20160004), 2018 Science and Technology Talents Boost-boosting Program of Shenzhen University General Hospital (SUGH2018QD029).

## Conflict of Interest

The authors declare that the research was conducted in the absence of any commercial or financial relationships that could be construed as a potential conflict of interest.

## Publisher’s Note

All claims expressed in this article are solely those of the authors and do not necessarily represent those of their affiliated organizations, or those of the publisher, the editors and the reviewers. Any product that may be evaluated in this article, or claim that may be made by its manufacturer, is not guaranteed or endorsed by the publisher.

## Supplementary Material

Supplementary Figure 1 **(A–D)** real-time primary human NK cell cytolysis using the REAL-TIME Cell Analyzer (RTCA); **(E–I)** Median fluorescence intensity of surface activating receptors on NK(YTS) and Tumor cells. **(J, K)** Survival curves for panobinostat therapy in B16F10 and CT26 tumor models (n =7); **(L–O)** NK cell depletion (ΔNK) one day before tumor inoculation. Assessment of mice body weight and Tumor images for **(L, M)** B16F10, **(N, O)** RMA-S tumor models.

Supplementary Figure 2 **(A)** Gating strategy for expanded primary human NK cells **(B)** Gating strategy for mice splenocyte-derived NK cells.

Supplementary Table 1 **(A)** Adhesion molecules primer sequences. **(B)** Tight junction related gene set primer sequences.
