# Supplementary figures and images for: Synergistic Tumor Cytolysis by NK Cells in Combination With a Pan-HDAC Inhibitor, Panobinostat
Source: Front Immunol. 2021 Aug 31;12:701671. doi: 10.3389/fimmu.2021.701671 (PMC8438531; doi:10.3389/fimmu.2021.701671)

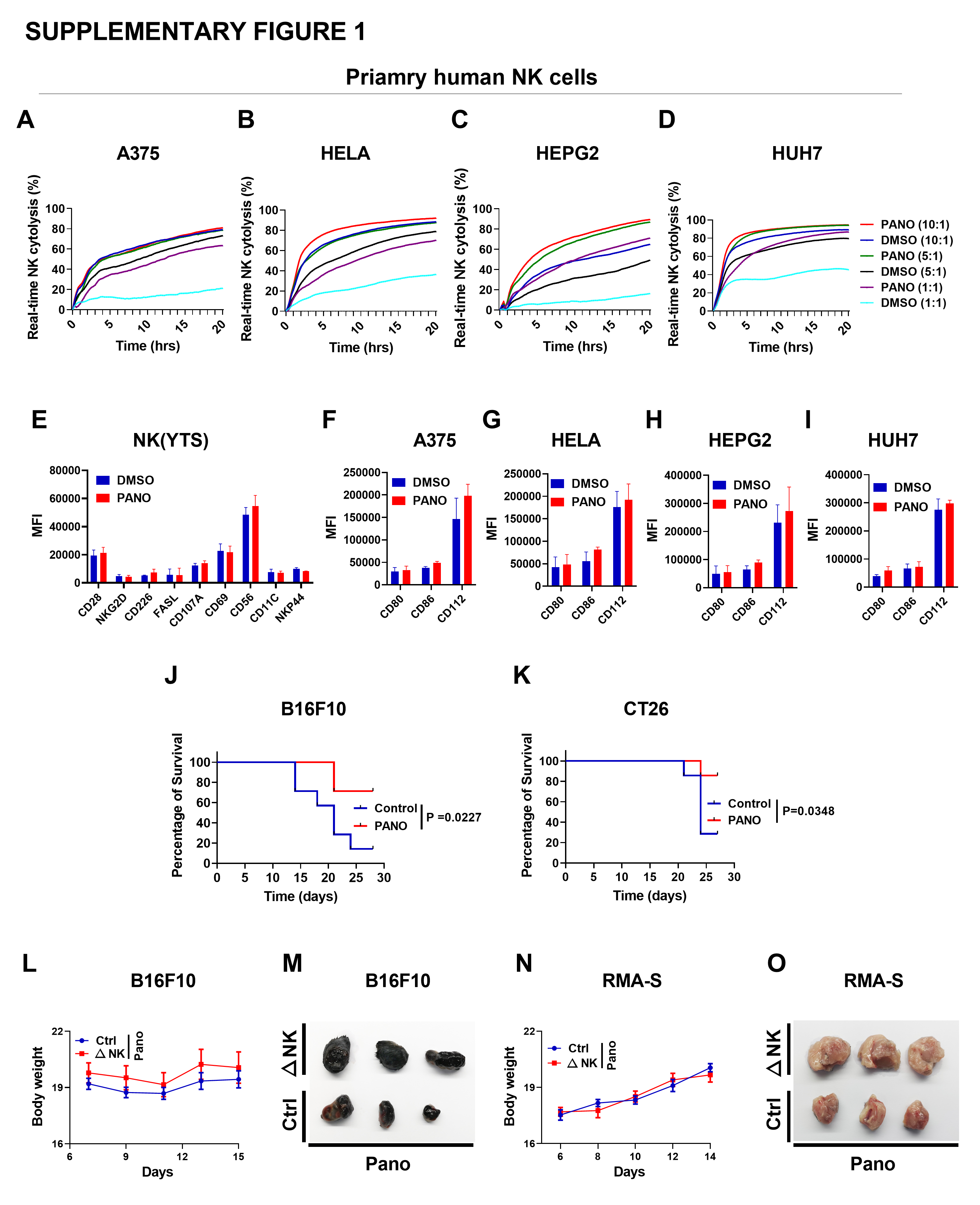

Supplement: Supplementary Figure 1 — (A–D) real-time primary human NK cell cytolysis using the REAL-TIME Cell Analyzer (RTCA); (E–I) Median fluorescence intensity of surface activating receptors on NK(YTS) and Tumor cells. (J, K) Survival curves for panobinostat therapy in B16F10 and CT26 tumor models (n =7); (L–O) NK cell depletion (ΔNK) one day before tumor inoculation. Assessment of mice body weight and Tumor images for (L, M) B16F10, (N, O) RMA-S tumor models. [file Image_1.tif]

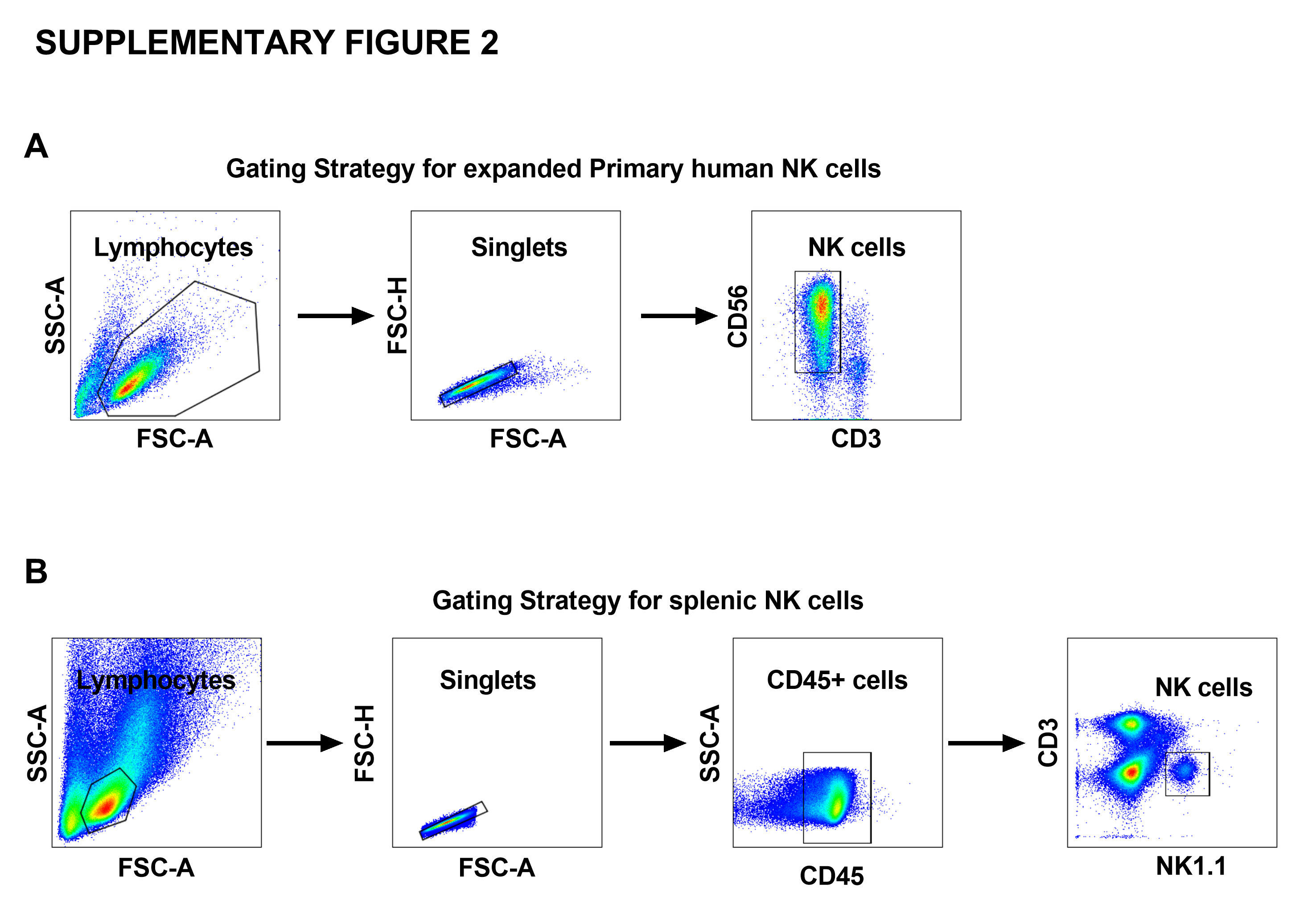

Supplement: Supplementary Figure 2 — (A) Gating strategy for expanded primary human NK cells (B) Gating strategy for mice splenocyte-derived NK cells. [file Image_2.tif]
