# Supplementary material for: Synergistic Tumor Cytolysis by NK Cells in Combination With a Pan-HDAC Inhibitor, Panobinostat
Source: Front Immunol. 2021 Aug 31;12:701671. doi: 10.3389/fimmu.2021.701671 (PMC8438531; doi:10.3389/fimmu.2021.701671)
Supplement: Supplementary Table 1 — (A) Adhesion molecules primer sequences. (B) Tight junction related gene set primer sequences. [file Table_1.docx]

**Synergistic tumor cytolysis by NK cells in combination with a pan-HDAC inhibitor, Panobinostat**

**Running title:** Synergistic tumor cytolysis by NK cells and Panobinostat

**Lukman O. Afolabi ^a, c, †^, Jiacheng Bi ^a, b, c, †^, Xuguang Li** **^d^, Adeleye O. Adeshakin** **^a, c^, Funmilayo O. Adeshakin ^a, c^, Haisi Wu ^a, c^,** **Dehong Yan** **^a, c^, Liang Chen ^a, c^, Xiaochun Wan ^a, c, *^**

^a^ Guangdong Immune Cell therapy Engineering and Technology research center, Center for Protein and Cell-based Drugs, Institute of Biomedicine and Biotechnology, Shenzhen Institutes of Advanced Technology, Chinese Academy of Sciences, Shenzhen, 518055, PR China

^b^ CAS Key Laboratory of Quantitative Engineering Biology, Shenzhen Institute of Synthetic Biology, Shenzhen Institutes of Advanced Technology, Chinese Academy of Sciences, Shenzhen 518055, China.

^c^ University of Chinese Academy of Sciences, Beijing, 100864, PR China

^d^ Department of Stomatology, Shenzhen University General Hospital, Shenzhen University Clinical Medical Academy, 1098 Xuan Yuan Road, Nanshan District, Shenzhen, Guangdong Province, 518055 China

**^†^** These authors have contributed equally to this work.

* Corresponding author. Xiaochun Wan.

E-mail address: [xc.wan@siat.ac.cn](mailto:xc.wan@siat.ac.cn)

**Supplementary Table 1**

**Table 1A: Adhesion molecules primer sequences**

| Target genes | Forward primer | Reverse primer |
| --- | --- | --- |
| CLDN9 | TGTGGAGGACGAAGGTGCCAAG | AGAAGTCCTGGATGATGGCGTG |
| NFASC | GGCAAAGCTGAAAACCAAGTCCG | TTCAGCCAGGAGACGGTGAGTT |
| CDH15 | AGCGTATCCGAGAACCACAAGC | CTGGATGCTGTAGATGACGCTG |
| NRXN2 | GACAATGAGTGGCACACGGTGA | CCGTCTCAATGTTGTGGAACTCC |
| SPN | GCAACCAGTCATCCTGCTGTTC | CTGGTCCTACTGGAGGTTTCTG |
| NCAM1 | CATCACCTGGAGGACTTCTACC | CAGTGTACTGGATGCTCTTCAGG |
| CNTN1 | GCTGGAAGATACACATGCACTGC | GTCTGAACCACGGCTCCAAGTA |
| ESAM | CGCTGTCCAATACCAGTGGGAT | CCTTGCAGACATAGACTCCAGC |
| ITGAL | CTGCTTTTGCCAGCCTCTCTGT | GCTCACAGGTATCTGGCTATGG |
| CD40 | CCTGTTTGCCATCCTCTTGGTG | AGCAGTGTTGGAGCCAGGAAGA |

**Table 1B: Tight junction related gene set primer sequences**

| Target genes | Forward primer | Reverse primer |
| --- | --- | --- |
| TJP3 | GCTTCCTCAAGGGCAAGAGCAT | CGTGTCAGGTTCTGGAATGGCA |
| ACTN3 | TCCAGCACCTGGCTGAGAAGTT | CACCTCCTGTAGCAAAGCCGAA |
| LLGL2 | TGCTGACACCTACCTGAAGGAC | ACAGGCTCATCCATTCTCCGCT |
| PARD6A | CTATACGGATGCTCATGGCGAC | GAGAGTTGGAGGCAAAAGCCAG |
| PRKCG | CCGCCTGTATTTCGTGATGGAG | CGATAGCGATTTCTGCCGCGTA |
| PRKCB | GAGGGACACATCAAGATTGCCG | CACCAATCCACGGACTTCCCAT |
| ACTN2 | GAGGGCAAGATGGTGTCGGATA | CTTCTCAGCCAGGTGTTCCAAG |
| RRAS | CTGCTGGTGTTCGCCATTAACG | GATCTGCCTTGTTCCCGACCAA |
| CLDN19 | AGTGCAAGCTCTACGACTCGCT | CACTTCATGCCAACTACGCTGAG |
| BETA ACTIN | CACCATTGGCAATGAGCGGTTC | AGGTCTTTGCGGATGTCCACGT |
